# Comment on ‘A point prevalence survey on antibiotic dispensing practices identifies targets for quality improvement of antimicrobial stewardship in community pharmacies in Dar es Salaam, Tanzania’

**DOI:** 10.1080/20523211.2026.2715838

**Published:** 2026-08-28

**Authors:** Ayman M. Mustafa, Shkar Mariwan Ahmed, Ari Jamal Hassan

**Affiliations:** a Kscien Organization for Scientific Research (Middle East Office), Sulaymaniyah, Iraq; b Medical Laboratory Department, College of Health Science, University of Human Development, Sulaymaniyah, Iraq; c Department of Pharmacy, College of Pharmacy, University of Human Development, Sulaymaniyah, Iraq; d Department of Nursing, College of Nursing, University of Human Development, Sulaymaniyah, Iraq

Dear Editor,

We read with great interest the point prevalence survey by Migiro and colleagues on antibiotic dispensing practices in community pharmacies in Dar es Salaam. The authors interviewed 500 exiting clients across 100 pharmacies selected by probability proportional to size across the city’s five municipalities, and characterised the proportion of attendees dispensed antibiotics, the prescription status of those products, the presumed indications, the source of the recommendation, and the distribution of dispensed agents across the World Health Organization (WHO) AWaRe categories. Their central observation – that roughly two thirds of attendees received antibiotics and that most did so without a prescription, frequently on the dispenser’s own recommendation – is an important and policy-relevant contribution, and their decision to recruit real attendees rather than simulated clients is a genuine strength that distinguishes this work from much of the earlier Tanzanian literature (Migiro et al., 2026). We congratulate the authors on a clearly written and timely study. Alongside these merits, and in a constructive spirit, we would welcome clarification on a few methodological and reporting matters that we believe would further strengthen the interpretation of the findings.

Our first point concerns the measurement instrument. The authors describe the questionnaire as ‘validated’ and note that it was adapted from Nabeel et al. and pilot-tested with ten participants to assess clarity, flow, and sequencing. Adapting an instrument that was developed in a different setting (Lahore, Pakistan) for use in Dar es Salaam necessarily introduces changes in language, health-system context, and locally available products, and it is generally recommended that such cross-cultural adaptation be accompanied by a fresh appraisal of content validity rather than an assumption that validity transfers intact with the items (Sousa & Rojjanasrirat, 2011). It would help readers if the authors could indicate whether content validity was formally quantified – for instance, through item – and scale-level Content Validity Indices (I-CVI and S-CVI) obtained from an expert panel, which have become a standard means of documenting that items adequately represent the construct of interest (Polit & Beck, 2006; Zamanzadeh et al., 2015). Relatedly, the pilot described appears to have addressed comprehension and layout but not the instrument’s reliability. A brief statement of internal consistency (for example, Cronbach’s alpha for any multi-item scales) or, where the tool is largely single-item and factual, a note on inter-interviewer or inter-rater agreement, would reassure readers that the data were captured consistently across the several trained assistant researchers who conducted the interviews (Tavakol & Dennick, 2011). Making these psychometric details explicit would substantiate the ‘validated’ descriptor and would allow other groups in the region to reuse the tool with confidence.

Second, we would offer a thought on the analytical framing of the factors associated with dispensing. The multivariable modified Poisson model appropriately estimates prevalence ratios, but it takes ‘being dispensed an antibiotic’ (yes/no) as its outcome, and this outcome combines two rather different situations – clients obtaining an antibiotic that had been appropriately prescribed, and clients obtaining one without a prescription or on a dispenser’s suggestion (Barros & Hirakata, 2003). Because the study’s central concern is non-prescription and potentially inappropriate dispensing, the associations reported with education, district, and occupation describe who is likely to receive any antibiotic rather than who is likely to receive one outside the intended regulatory pathway. We wonder whether the authors considered modelling non-prescription dispensing specifically as the outcome, or presenting it as a stratified analysis, which would align the regression more closely with the stewardship question and would help distinguish, for example, whether the higher dispensing observed among clients with technical or vocational education reflects greater self-medication or simply a greater burden of infection. A related nuance is that appropriateness was inferred largely from prescription status and guideline lists rather than from an independent clinical assessment of whether each antibiotic was indicated; and since 30% of recipients cited a hospital visit as the source of their recommendation, it is possible that some clients recorded as receiving antibiotics ‘without a prescription’ were in fact continuing a course initiated during a prior consultation. A brief note on how these situations were handled would help readers calibrate how much of the observed dispensing is truly discordant with good practice.

Third, and on a related note, we would gently encourage a more complete presentation of the WHO AWaRe analysis, which underpins much of the stewardship argument. The reported categories – Access 56% (201/356), Watch 32% (115/356), and Reserve 3% (10/356) – together account for 326 of the 356 antibiotics dispensed, leaving approximately 30 products (about 8%) unclassified in the text. The AWaRe classification comprises four groups, the fourth being the ‘Not recommended’ fixed-dose combinations, and it is plausible that the unaccounted products include the ampicillin – cloxacillin combination that the authors themselves report as frequently dispensed (28/356), largely for respiratory symptoms (Sharland et al., 2018; World Health Organization, 2022). If that is the case, this residual is not a trivial rounding artefact but a stewardship-relevant finding in its own right, since ‘Not recommended’ combinations are precisely the products that the AWaRe framework discourages. Reporting all four categories, and clarifying how combination products (and any topical or non-antibacterial agents) were handled within the denominator of 356, would give a fuller and internally consistent picture of dispensing quality and would sharpen the comparison the authors draw against the 70% Access target. We offer these comments in the hope that they are useful, and we thank the authors for a valuable addition to the Tanzanian evidence base on community pharmacy dispensing.
